# Language transfer in L2 academic writings: a dependency grammar approach

**DOI:** 10.3389/fpsyg.2024.1384629

**Published:** 2024-05-09

**Authors:** Yude Bi, Hua Tan

**Affiliations:** ^1^Fudan University, Shanghai, China; ^2^Central China Normal University, Wuhan, China

**Keywords:** dependency distance, English L2 academic writing, native language transfer, corpus analysis, dependency direction

## Abstract

Dependency distance (DD) is an important factor in language processing and can affect the ease with which a sentence is understood. Previous studies have investigated the role of DD in L2 writing, but little is known about how the native language influences DD in L2 academic writing. This study is probably the first one that investigates, though a large dataset of over 400 million words, whether the native language of L2 writers influences the DD in their academic writings. Using a dataset of over 2.2 million abstracts of articles downloaded from Scopus in the fields of Arts & Humanities and Social Sciences, the study analyzes the DD patterns, parsed by the latest version of the syntactic parser Stanford Corenlp 4.5.5, in the academic writing of L2 learners from different language backgrounds. It is found that native languages influence the DD of English L2 academic writings. When the mean dependency distance (MDD) of native languages is much longer than that of native English, the MDD of their English L2 academic writings will be much longer than that of English native academic writings. The findings of this study will deepen our insights into the influence of native language transfer on L2 academic writing, potentially shaping pedagogical strategies in L2 academic writing education.

## 1 Introduction

Academic writing in a second language (L2) poses many challenges for L2 learners. One important aspect of academic writing quality is syntactic complexity (Lu, 2011), which can be measured by dependency distance (DD)—the linear distance between syntactically related words in a sentence (Tesnière, 1959; Liu, 2007, 2008; Hudson, 2010; Liu et al., 2017, 2022). In their exploration of DD, researchers have also examined dependency direction (Liu et al., 2009; Liu, 2010; Jiang and Liu, 2015; Wang and Liu, 2017; Fan and Jiang, 2019)—a concept that delineates the positional relationship between a governor and its dependent within syntactically connected word pairs, specifically whether the governor appears after or before its dependent. DD has been recognized as a valid measure of syntactic complexity and language comprehension difficulty (Liu, 2008; Oya, 2011). Research has found that writers tend to minimize DD in the writings with their native languages (Temperley, 2007, 2008; Futrell et al., 2015; Temperley and Gildea, 2018; Lei and Wen, 2019; Lu and Liu, 2020), resulting in more locally coherent sentences. However, less is known about the DDs of English L2 academic writings and whether the writers’ native languages influence the DD of their L2 academic writings.

This study intends to investigate whether the DDs of English L2 academic writing are affected by the writers’ native languages. Specifically, it compares the DDs of the abstracts of journal articles written by English L2 users and English native speakers. English is found to be different from other languages, like French, Spanish, Korean, and Arabic, in thought patterns and rhetorical structures (Kaplan, 1966), which may impact the DDs in L2 writing. However, L2 writing may also be shaped by universal pressures for efficient processing, driving DDs toward a common optimal range (Liu et al., 2017) (See section 2 for further explanation).

To test these accounts, we analyzed the DDs of English academic writings by English L2 users and native speakers. We extracted the DDs from each text using syntactic parsing and compared the distributions statistically. This allows us to determine if native language background influences DD in English L2 academic writing, shedding light on how linguistic backgrounds impact L2 syntactic structures in academic writing. Such insights could contribute significantly to our understanding of language acquisition and the challenges faced by individuals writing in an L2 academic context. The findings will have implications for understanding the role of native language transfer in English L2 writing.

### 1.1 Previous research

Syntactic complexity, which involves the range and sophistication of syntactic structures, is considered a key dimension of academic writing development and quality (Ortega, 2003; Lu, 2011). A quantitative metric that has garnered heightened attention in syntactic complexity research is DD. DD offers an index for evaluating the density or dispersion of grammatical connections throughout a text. Research suggests that dependency distance minimization (DDM) reflects a universal cognitive pressure for efficient human information processing and linguistic production (Futrell et al., 2015). English writers have been found to prefer syntactic structures with shorter dependencies to reduce integration difficulty and yield more locally coherent sentences (Temperley, 2007). However, cross-linguistic differences have also been observed, with head-final languages like Japanese, Korean, and Turkish showing greater distances attributable to word order variation (Futrell et al., 2015). Chinese and English show different dynamic valency of words and syntactic dependency structures (Lu et al., 2018). Liu et al. (2009) also found that Chinese shows quite different features in dependency relations, with its dependencies tending to be governor-final and mean dependency distance (MDD) being much higher than languages like English, German, and Japanese. While research has examined DDs in native language writing, fewer studies have investigated DDs in L2 academic writing.

### 1.2 DD optimization in L1 academic writing

Research consistently shows a strong tendency for compact, local syntactic structures in academic writing by L1 writers, which is argued to reflect pressures for efficient linguistic processing and production (Liu et al., 2017). An early study by Temperley (2007) analyzed DDs in the Wall Street Journal portion of the Penn Treebank. It is found that writers favor structures with shorter dependencies, which is evidenced by their preference for short left-branching constituents. Temperley argued that writers optimize and minimize dependency lengths to yield more incrementally interpretable sentences to facilitate comprehension. Futrell et al. (2015) also concluded that DDM is a universal characteristic across human languages, suggesting that variation in language can be explained by the general properties of human information processing. The authors argue that minimizing DDs enhances the efficiency of parsing and producing natural language, reducing integration costs and enabling more efficient packing of information into sentences. Lu and Liu (2020) also discovered a tendency of DDM within noun phrases, potentially due to limitations in human working memory capacity.

However, cross-linguistic differences have also been observed, attributable to syntactic variations across languages. Futrell et al. (2015) found that head-final languages like Japanese, Turkish, and Korean show much less DDM than head-initial languages like English, Italian, and Indonesian. Temperley and Gildea also found great differences across languages in DD. Their study confirms that DDM serves as an important factor in language structure and cognition, which is evidenced by the fact that writers and speakers tend to prefer structures that reduce dependency length when a language allows for different orderings of constituents (Temperley and Gildea, 2018). Nonetheless, Futrell et al. (2015) argue that DDM remains a universal quantitative property, as overall DDs were substantially shorter than random baselines (benchmarks created by randomly reorganizing the head word and its dependents in dependency trees, without following any specific linguistic word order rules), across all 37 diverse languages in their study. They contend that despite the structural variations among languages influencing their DDs, there is a universal aim in all languages to minimize DDs for the sake of efficiency, within the bounds of their structural limitations. The following example demonstrates the impact of syntactic variations on DD, with the specifics of the dependency relations delineated in Table 1.

**Table 1:** Dependency relations of Examples 1a and b.

Dependent ID	Token	Part of Speech	Governor ID	Dependency Relation	Dependency Distance
1	John	NNP	2	nsubj	1
2	threw	VBD	0	ROOT	/
3	out	RP	2	compound: prt	1
4	the	DT	6	det	2
5	old	JJ	6	amod	1
6	trash	NN	2	obj	4
7	sitting	VBG	6	acl	1
8	in	IN	7	case	1
9	the	DT	10	det	1
10	kitchen	NN	8	obl	2

Dependent ID	Token	Part of Speech	Governor ID	Dependency Relation	Dependency Distance
1	John	NNP	2	nsubj	1
2	threw	VBD	0	ROOT	/
3	the	DT	5	det	2
4	old	JJ	5	amod	1
5	trash	NN	2	obj	3
6	sitting	VBG	5	acl	1
7	in	IN	6	case	1
8	the	DT	9	det	1
9	kitchen	NN	7	obl	2
10	out	RP	2	compound: prt	8

Example 1. a: John threw out the old trash sitting in the kitchen. b: John threw the old trash sitting in the kitchen out (Futrell et al., 2015: 10337).

The two sentences above convey the same concept and have identical structures, except for the positioning of the word “out.” However, their total and mean DDs significantly differ, at 14 vs. 20 and 1.55 vs. 2.22, respectively. The varying DDs require distinct cognitive effort and working memory capacity. Example 1b where “out” is moved to the end demands more cognitive effort and working memory. For lower cognitive effort and working memory load, Example 1a, which is free from particle movement and exemplifies DDM, is preferred.

In sum, research shows syntax is optimized for brevity both within and across languages to aid production and comprehension, but differences exist due to language-specific conventions.

### 1.3 DDs in L2 academic writing

While numerous studies have examined L1 DDs, few studies have investigated DDs in L2 academic writing. Ouyang et al. (2022) investigated the writing proficiency of beginner, intermediate, and advanced learners by DD measures. They discovered that the MDD overall is significantly effective at distinguishing between each pair of consecutive proficiency levels. Hao et al. (2022) verified the application of DD and its probability distribution as syntactic indicators of English as interlanguage from the perspective of language typology. They found that the MDDs of L2 learners with different backgrounds of native language gradually approach that of the target language with the improvement of their L2 proficiency. Li and Yan (2021) similarly found that MDD can serve as an effective indicator to measure the syntactic complexity of Japanese EFL learners’ interlanguage. Similarly, Hao et al. (2023) found in their study that dependency parameters have universal applicability in reflecting interlanguage proficiency.

To date, very few studies have directly compared the DD profiles of L2 writers from different L1 backgrounds composing in the L2. Gao and He (2023) examined the MDD of Ph.D. dissertation abstracts written by L1 (native English) and L2 (English as a foreign language) academic writers across language backgrounds and disciplines, finding that MDD successfully distinguishes between academic texts from various linguistic backgrounds and disciplines. They argued that the authors’ efforts to make comprehension easier for readers result in the shorter MDD observed in physics and chemistry abstracts.

Overall, research on L2 DDs remains limited, with very few studies comparing profiles of different L1 groups in natural academic writing tasks. In particular, few studies examined whether L2 DDs and dependency directions are influenced by L2 writers’ native language. This represents a significant gap, as investigating cross-linguistic differences can elucidate the role of L1 transfer versus universality in L2 syntactic development, with key theoretical and pedagogical implications (Ortega, 2003).

### 1.4 Transfer of syntactic features in L2 acquisition

In examining the impact of native language transfer in L2 acquisition, scholars have extensively investigated how syntactic features influence L2 language production. Whong-Barr and Schwartz (2002) reveal that in the L2 acquisition process, children’s mastery of English dative constructions is significantly shaped by their native linguistic backgrounds, underscoring the influence of L1 syntactic frameworks and prevalent overgeneralization patterns on their learning trajectory. Chan (2004) demonstrates evidence of syntactic transfer from Chinese to English among Hong Kong Chinese ESL learners, revealing that learners often think in Chinese before writing in English, leading to interlanguage structures that closely resemble or mirror the syntactic patterns of their first language, particularly in complex target structures and among learners of lower proficiency levels. Recent advancements in second language acquisition research have introduced theories such as the Interpretability Hypothesis by Tsimpli and Dimitrakopoulou (2007), which argues that learners can acquire L2 features interpretable across syntax and other cognitive systems like semantics or pragmatics, regardless of their presence in L1; the Interface Hypothesis by Sorace and Filiaci (2006), highlighting the particular difficulties learners face with language elements that integrate syntax with semantics or discourse; and the Feature Reassembly Hypothesis by Lardiere (2009), emphasizing the primary challenge of reconfiguring L1 features to conform to the target language’s system, often leading to substantial learning challenges, especially where the languages’ feature systems notably diverge.

Though these new theories have been much discussed in recent years, language transfer theory remains relevant due to its powerful explanatory capabilities, able to account for many phenomena in L2 acquisition. This study aims to explore syntactic transfer through the lens of DG. We contend that the dependency patterns of an individual’s native language can influence those in their L2 writings. For instance, consider Chinese, which is predominantly a head-final language, and English, primarily a head-initial language. The MDD of Chinese stands at 3.662, markedly higher than English’s MDD of 2.543. We hypothesize that this significantly larger MDD in Chinese will lead to extended MDDs in L2 English writings by Chinese learners, as a consequence of language transfer. This study will verify our hypothesis.

## 2 Objectives and significance

This study delves into how L1 backgrounds influence L2 writing, particularly focusing on DDs in academic writing. It examines whether different L1 backgrounds result in distinct DD patterns in English L2 writing, potentially due to L1 transfer, or if universal linguistic principles lead to uniform patterns across L1 groups. This inquiry aims to illuminate key debates within second language acquisition (SLA) regarding the influence of native language versus universal syntax principles. It seeks to fill significant gaps in existing research and enhance writing instruction practices by clarifying the extent of cross-linguistic influence versus universal principles in L2 writing development.

The outcomes of this research could provide significant implications for both SLA theory and academic writing instruction. By identifying whether L2 writers’ dependency profiles are shaped more by their L1 syntax or universal syntax norms, this study inform educational strategies—determining if writing instruction should be tailored to specific L1 backgrounds or aligned with broader, universal writing strategies. These insights will guide educators on whether to prioritize language-specific strategies or general methods to help L2 writers reach native-like proficiency.

## 3 Theoretical framework

This study investigates the effect of native language on English L2 academic writing, employing quantitative analysis of dependency grammar (DG). DG, serving as a theoretical linguistic framework, delineates language structure by scrutinizing the relationships among its components. These relationships, known as dependencies, are asymmetrical connections between two constituents of a sentence, typically words, where one assumes the role of the governor or head, and the other, the dependent or modifier (Fraser, 1994). In DG, DD and dependency direction, often utilized as variables in linguistic studies, serve as two critical indices for quantitative analysis. DD (Liu, 2007, 2008; Liu et al., 2017), also known as dependency length (Temperley, 2007, 2008; Gildea and Temperley, 2010; Futrell et al., 2015; Temperley and Gildea, 2018), refers to the linear positional difference between two words within a sentence serving as governor and dependent (Hudson, 1995, 2010; Liu et al., 2009). It is measured by the number of intervening words between dependents and their governors (Hudson, 1995). For any dependency relation between two words *Wx* and *Wy*, if *x* is the governor and *y* is its dependent, their DD equals the difference *x* − *y*; thus, adjacent words have a DD of 1. A positive distance signifies that the governor follows the dependent, whereas a negative distance indicates the governor precedes the dependent. Nevertheless, for the calculation of MDD, the absolute value of DD is used. The MDD for a sentence can be determined using the equation below:


(1) $$\mathrm{MDD}\;\left(\mathrm{sentence}\right)=\frac{1}{n-1}\overset{n-1}{\underset{i=1}{\sum }}|DD_{i}|$$

where *n* represents the total number of words in a sentence and DD*i* indicates the DD of the *i*-th syntactic relation within the sentence (see Liu et al., 2009: 166). Typically, there exists one word in each sentence that does not have a governor. This word is termed the root verb, and its DD is considered zero.


(2) $$\mathrm{MDD}\;\left(\mathrm{sample}\right)=\frac{1}{n-s}\overset{n-s}{\underset{i=1}{\sum }}|DD_{i}|$$

where *n* represents the total number of words in a sample and *s* indicates the number of sentences in the sample (see Liu et al., 2009: 166). DD*i* refers to the DD of the *i*-th syntactic relation within the sample.

**Figure 1:**
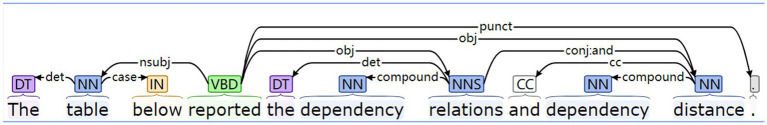
Dependency structure of Example 2.

The example below illustrates a dependency analysis. Figure 1 lists the dependency structure of Example 2, while Table 2 details the dependency relations and distances associated with it.

**Table 2:** Dependency relations of Example 2.

Dependent id	Token	Part of speech	Governor id	Dependency relation	Dependency distance
1	The	DT	2	det	1
2	table	NN	4	nsubj	2
3	below	IN	2	case	1
4	reported	VBD	0	ROOT	/
5	the	DT	7	det	2
6	dependency	NN	7	compound	1
7	relations	NNS	4	obj	3
8	and	CC	10	cc	2
9	dependency	NN	10	compound	1
10	distance	NN	7	conj	3
11	.	PUNCT	4	punct	/

Example 2. The table below reported the dependency relations and dependency distance.

Figure 1 depicts the dependency relations between governors and their dependents in the example sentence. Syntactically related word pairs are connected by labeled lines with arrows pointing from the governor to the dependent. These labels, including *nsubj*, *det*, *obj*, *conj*, and *punct*, denote the specific dependency relations between the connected words.

Based on Eq. 1, the MDD of the example is:


$$\mathrm{MDD}\;\left(\mathrm{Example}\;2\right)=\frac{|1+2+1+2+1+3+2+1+3|}{9}=1.7778$$

As mentioned before, DD can manifest as positive or negative contingent on whether the governor precedes or succeeds its dependent, thereby indicating the direction of dependency. When the governor precedes its dependent, DD is negative, indicating a governor-initial dependency relation, otherwise positive, denoting a governor-final dependency relation. The dependency direction within a sample can be quantified by calculating the percentages of governor-initial (or head-initial) and governor-final (or head-final) relations, using the following equations:


(3) $$\begin{array}{l} \mathrm{Percentage of head}-\mathrm{final dependency}\\ \;\;\;\;\;=\frac{\mathrm{frequencies of the head}-\mathrm{final dependency}}{\mathrm{total number of dependencies in the treebank}}\times 100 \end{array}$$

(4) $$\begin{array}{l} \mathrm{Percentage of head}-\mathrm{initial dependency}\\ \;\;\;\;=\frac{\mathrm{frequencies of the head}-\mathrm{initial dependency}}{\mathrm{total number of dependencies in the treebank}}\times 100 \end{array}$$

(see Liu, 2010: 1570)

Applying the aforementioned Eqs. 3 and 4, the dependency direction of Example 2 is:


$$\mathrm{Percentage of head}-\mathrm{final dependency of Example}\;1=\frac{6}{9}\times 100=66.7\%$$

$$\mathrm{Percentage of head}-\mathrm{initial dependency of Example}\;1=\frac{3}{9}\times 100=33.3\%$$

Evidently, the example sentence contains substantially more head-final dependencies compared to head-initial ones, indicating that most dependents precede their governors.

This study primarily focuses on the overall differences or similarities in DD between L1 and L2 English, without delving into the specific types of dependencies in each language. Our aim is to investigate the broad impact of native language on L2 writing, rather than examining the nuanced differences in dependency types and their respective DDs. These finer details of dependency types and corresponding DDs will be the subject of our future research.

This study’s DG analysis benefits from recent advances in natural language processing (NLP) technology, particularly in automating part-of-speech tagging and syntactic parsing. Previously, the slow and costly manual processes hindered the development of treebanks—key resources containing tagged and parsed sentences. However, modern NLP has overcome these challenges by enabling automatic tagging and parsing via machine learning, leveraging existing treebanks. These developments have expanded the application of NLP across the humanities, situating this research within the broader trend of integrating NLP into linguistic studies.

## 4 Methodology

### 4.1 Research questions

In the present study, we intend to answer the following three questions:

1. Is there a significant difference in DD between English L2 academic writings and native academic writings?
2. Is there a significant difference in dependency direction between English L2 academic writings and native academic writings?
3. Is the DD of L2 academic writings influenced by native languages?

### 4.2 Data collection

The data for the present study was sourced from Scopus, selected for its status as the most extensive academic database and its established reliability as a data collection source across numerous studies (Crosthwaite et al., 2022, 2023; Zakaria and Aryadoust, 2023). Articles were chosen from the disciplines of Arts and Humanities and Social Sciences, limiting the selection to the “article” type while excluding “book review” and “book chapter” types, with an additional restriction for language to English only. In exporting the articles, we included the complete metadata for each article. Altogether over 2.65 million articles were extracted and stored in CSV files, with each metadata item allocated to a distinct column. Articles originating from various countries were segregated into separate CSV files, amounting to 178 files in total. The methodology for cleaning and processing the raw data is outlined in the following section.

### 4.3 Data cleaning and processing

We cleaned and processed the raw data using the following procedures. First, we wrote an R script to extract the “abstract” and “affiliation” columns. Second, we extracted the country names from the affiliation column, removed rows in the abstract column where no abstract was available using an R script, and manually checked the rows in the affiliation column where the country names were not available. Following the cleaning process, we obtained more than 2.22 million abstracts, totaling over 408.9 million tokens. Next, we calculated the dependency distance and direction for each abstract in each CSV file based on Eq. 2. This calculation was performed in Python, utilizing the Stanford CoreNLP package version 4.5.5. This package was selected due to its strong performance and established reliability as an NLP tool, as evidenced by various studies (Manning et al., 2014; Blšták and Rozinajová, 2022; Hashemi-Namin et al., 2023; He and Ang, 2023).

To categorize the dataset into L2 and native writings, we implemented a two-phase approach. In the first phase, we classified the abstracts according to the authors’ country of origin, identifying writings from the United Kingdom, United States, Australia, Canada, South Africa, New Zealand, Ireland, Bermuda, Jamaica, Trinidad and Tobago, Guyana, Barbados, and the Bahamas as native. In contrast, abstracts originating from any other country were designated as L2 writings. However, using affiliations to distinguish L2 from native writings is not entirely reliable, as L2 writers may study or work in countries where English is the primary language. To enhance the accuracy of classifying L2 and native writings, we introduced a second step involving a Python script that utilizes the nationalize.io API. This web-based service predicts nationalities from names using a vast database of names linked to their corresponding countries. We then compared the nationality predictions from nationalize.io with the initial phase’s results. In cases of discrepancies between the two sets of results, which were infrequent, we performed manual verification by consulting online sources to ascertain the authors’ nationalities. Through this dual-step approach, we significantly improved the precision of our classification between L2 and native writings. Despite this thorough double-check, there might still be a small number of cases where the classification was not accurate. However, given the vast size of our dataset, totaling over 2.22 million entries, these few discrepancies are unlikely to significantly impact our overall findings. Besides, other factors might affect the quality of L2 writing. The experiences of L2 writers, such as studying abroad or having their work edited by native speakers, could contribute to the subtleties of their writing. Nevertheless, we maintain that these factors do not substantially alter the fundamental linguistic characteristics of L2 writings. While there may be exceptional instances where they do, these are not expected to cause major deviations in our overall findings. Future research could consider incorporating these variables into their study designs to further enrich and complement our findings.

## 5 Results

### 5.1 Overall descriptive statistics

The table below presents the overall descriptive statistics of the data used in this study.

The overall descriptive statistics show that L2 writings have longer MDDs and higher percentages of governor-final dependencies (DDI) than native writings for both datasets. As the size of the datasets reached beyond the limit of Shapiro–Wilk and Student’s *t*-test, both of which require a sample size below 5,000, we used the Anderson-Darling normality test and Kolmogorov–Smirnov test to compare the native writings and L2 writings. The results show that native writings and L2 writings are significantly different in both MDD and DDI for both datasets (*p* < 0.0001), indicating that L2 writings have significantly longer MDDs and higher percentages of governor-final dependencies than native writings.

Based on the descriptive statistics, we can offer a positive answer to our research questions 1 and 2 regarding whether there is a significant difference in dependency distance and direction between English L1 and L2 academic writings. There is a significant difference in the MDD and DDI of the two groups. Yet, it is not sure whether the significant difference is influenced by native language transfer. In the next section, we will discuss it based on more detailed results.

### 5.2 MDD and DDI of English L2 academic writings with different language backgrounds

**Figure 2:**
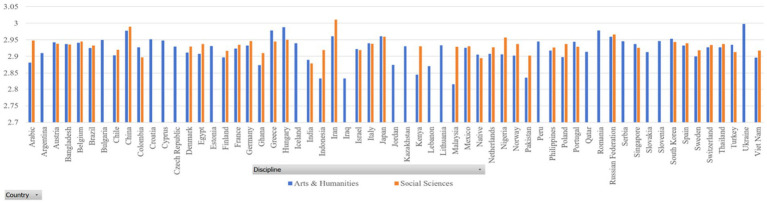
MDD of samples.

**Figure 3:**
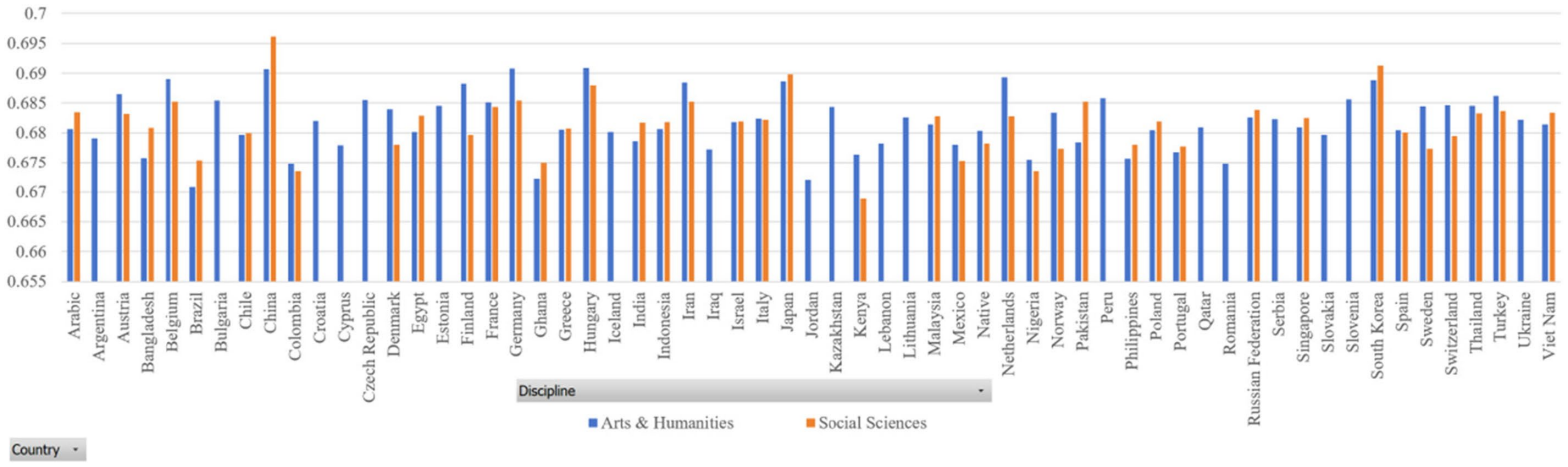
DDI of samples.

In our datasets, the sample size of some countries is very small (for example, Gambia and Guinea). Thus, we only selected those countries with a sample size of 500 and above. Figures 2, 3 report the dependency distances of the selected countries in the Arts and Humanities group and the Social Sciences group, respectively. (Detailed reports of the MDD and DDI are available upon request).

In Table 3, MDD(sd) is the standard deviation of MDD, DDI is the mean ratio of dependency relations where governors are preceded by their dependents, in other words, DDI is mean governor-final ratio, and DDI(sd) is the standard deviation of DDI. The results in both sub-datasets show that the MDDs of both English native and L2 academic writings are much longer than that of the MDD of English (2.543) according to Liu (2008). Liu’s calculation of the MDD of English is based on news texts. Our study examines the MDD of academic texts. News texts and academic texts are different genres showing different linguistic features. Their differences in MDD show that genre is a factor that affects MDD, which is partially in line with the findings of Wang and Liu (2017). According to their study, genre affects dependency distance and direction significantly, but the effect is very small. They hold that “dependency distance is primarily determined by universal cognitive factors rather than genre-specific stylistic factors” (Wang and Liu, 2017: 135). Yet, in our study, we find that English native academic writings have an MDD of 2.9, much larger than the MDD of English news texts, which is 2.543 (Liu, 2008). In Wang and Liu’s (2017) study, the ratio of dependency relations where governors precede their dependents is between 46 and 51%, while in our study, this ratio is around 33% as the ratio of governors following dependents is around 68%. This again shows that the genre of English academic writings has a much higher ratio of governor-final dependencies. Such a finding indicates that genre has a significant influence on MDD, at least in terms of the genre of academic writing. Yet, as we only examined one genre, it is not safe to claim that genre has a large effect on its influence over DD, which needs further investigation with samples from different genres.

**Table 3:** Overall descriptive statistics of the data.

Dataset		Number of abstracts	Tokens (total)	Tokens (mean)	MDD	MDD (sd)	DDI	DDI (sd)
Social Sciences	All	1,360,291	249,746,453	183.6	2.9081	0.3542	0.6805	0.0365
	Native writings	1,046,179	185,956,693	177.7	2.8950	0.3505	0.6782	0.0363
	L2 writings	314,112	63,789,760	203.1	2.9517	0.3628	0.6887	0.0360
Arts & Humanities	All	860,018	159,209,295	185.1	2.9159	0.3686	0.6823	0.1238
	Native writings	457,919	81,836,408	178.7	2.9055	0.3819	0.6804	0.0448
	L2 writings	402,099	77,372,887	192.4	2.9278	0.3525	0.6843	0.1746

We proceeded to make Mann–Whitney U tests between native writings and L2 writings with different language backgrounds for the two sub-datasets. Mann Whitney U test is chosen because the native group has a very large sample size and is not in a normal distribution. As the results of Mann–Whitney U tests include many pairs of comparison, which takes up much space, they are not reported here and are available upon request. The results reveal that English native academic writings are significantly different in both MDD and DDI from English L2 academic writings of different language backgrounds for both sub-disciplines, as the *p* values are all below the significance level (0.05), with large effect sizes (*R* > =0.8) for most pairs. This finding further confirms the result reported previously in Table 3 where English native academic writings are found to be significantly different from English L2 academic writings on the whole.

As the Mann–Whitney U test examines whether two samples come from the same population, but does not reveal the correlation between variables, we did correlation analyses to find whether the differences in MDD and DDI are related to the nature of the samples, that is, native academic writings or L2 academic writings, to explore whether the language backgrounds of English L2 academic writings affect their dependency distances and dependency directions. We made a binomial logistic regression in R by the basic function *glm* with the two levels of the abstract type, Native vs. L2 as the response variable and MDD and DDI as predictor variables. Besides, we did another two analyses, linear regression analysis in R by the basic function *lm* and correlation analysis in R by the basic function *cor.test*. The three analyses are made for mutual corroboration.

The results of the binomial logistic regression in Table 4 show a significant correlation between article type and MDD and DDI for both sub-datasets, with *p* values below the significance level (0.05). Since the article type is a binary categorical variable, native vs. L2, the strong correlation indicates that whether the article type is native or L2 has a significant influence over MDD and DDI.

**Table 4:** Binomial logistic regression analysis results.

Discipline	Variable	Estimate	Std. Error	*z*.value	p
Arts & Humanities	(Intercept)	−3.2263	0.0439	−73.4981	0.0000
	MDD	0.0429	0.0062	6.8887	0.0000
	DDI	4.3710	0.0607	71.9540	0.0000
Social Sciences	(Intercept)	−7.4244	0.0417	−177.8757	0.0000
	MDD	0.3766	0.0057	66.2590	0.0000
	DDI	7.4941	0.0575	130.2762	0.0000

The results of linear regression in Table 5 and correlation analyses in Table 6 both confirm the correlation between article type and MDD and DDI, with *p* values much lower than the significance level (0.05). As the three correlation-related analyses all show a significant influence of article type on MDD and DDI, it could be claimed that on the whole there is an effect of native language transfer on the MDD and DDI of English L2 academic writing. Currently, no study has been found to examine the MDD and DDI of all languages in the world due to various reasons, though some studies have investigated the DDs of some languages, such as Chen and Gerdes (2020), Jing and Liu (2017), Futrell et al. (2015) and Liu (2008). However, examining studies such as Liu (2008) reveals a trend: the greater the MDD in the background language of English L2 academic writings, the longer the MDD tends to be in English L2 academic writings themselves. For example, in the data of Social Sciences, L2 writings with Chinese as their background language have a much higher DD than the English native ones, with their MDD being 2.9896 vs. 2.8950, compared to the MDD of original Chinese and English, which is 3.662 vs. 2.543 (Liu, 2008). It is the same, for instance, with Hungarian, German, and Spanish, 2.9500 vs. 2.8950 and 3.446 vs. 2.543, 2.9464 vs. 2.8950 and 3.353 vs. 2.543, 2.9400 vs. 2.8950 and 2.665 vs. 2.543.

**Table 5:** Linear regression analysis results.

Discipline	Variable	Estimate	Std. Error	*t*.value	p
Arts & Humanities	(Intercept)	−0.2974	0.0108	−27.6003	0.0000
	MDD	0.0107	0.0015	6.9364	0.0000
	DDI	1.0798	0.0149	72.4425	0.0000
Social Sciences	(Intercept)	−0.8485	0.0071	−119.8052	0.0000
	MDD	0.0676	0.0010	66.5099	0.0000
	DDI	1.2973	0.0099	131.5301	0.0000

**Table 6:** Correlation analysis results.

Discipline	Variable	Cor_Coefficient	p	t	CI_Lower	CI_Upper
Arts & Humanities	MDD	0.0139	0.0000	12.8731	0.0118	0.0160
	DDI	0.0789	0.0000	73.2546	0.0768	0.0810
Social Sciences	MDD	0.0674	0.0000	78.8302	0.0658	0.0691
	DDI	0.1177	0.0000	138.2306	0.1160	0.1194

### 5.3 MDD and DDI of English L2 academic writings from different language families

To further confirm or examine the effect of native language transfer on the MDD and DDI of English L2 academic writing, we categorized the language backgrounds into several groups based on the classification of language families by Katzner (2002) and Brown and Ogilvie (2009). Then, we calculated the MDD and DDI of the English L2 academic writings of different language background groups and made Mann–Whitney U test.

**Figure 4:**
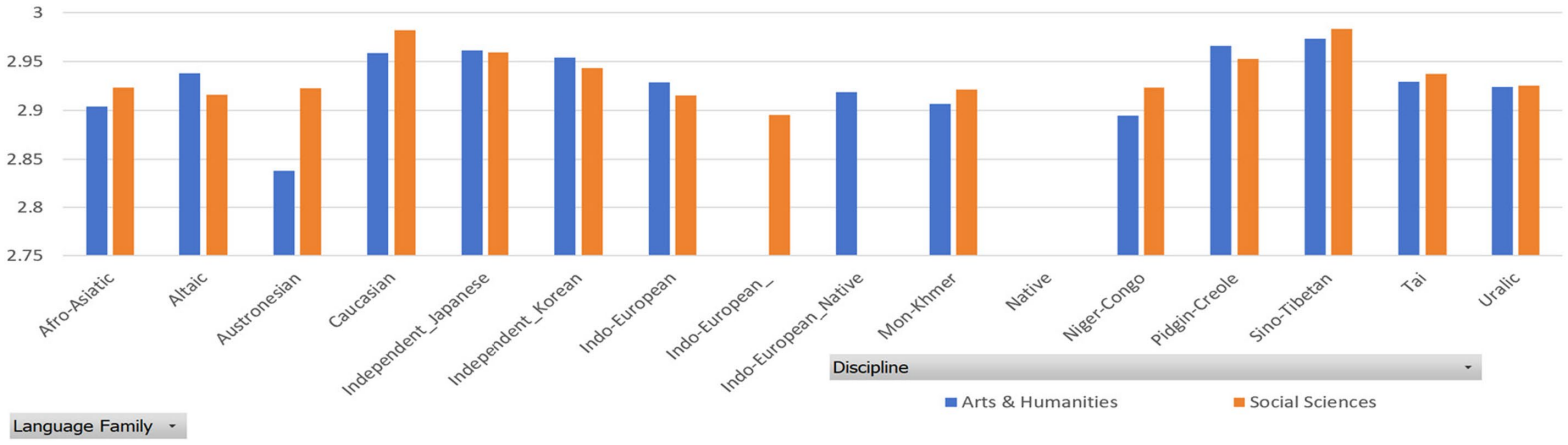
MDD of samples grouped by language family.

**Figure 5:**
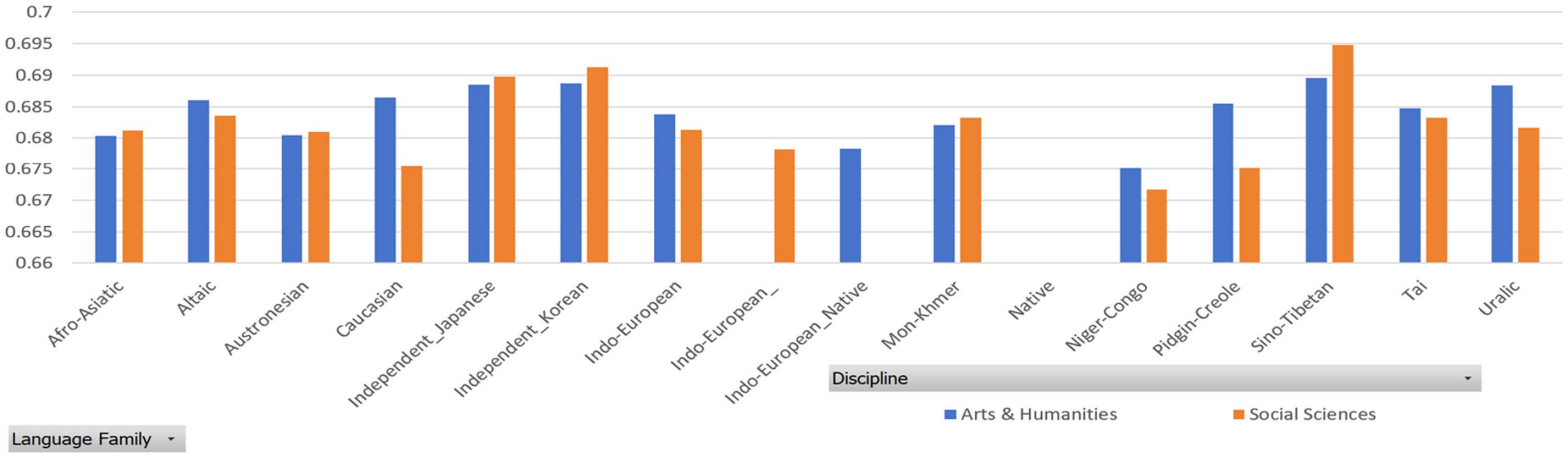
DDI of samples grouped by language family.

Tables 7, 8 report the MDD and DDI of the samples grouped by language family, which are accompanied by Figures 4, 5 for better visualization. The results show that English native academic writings are significantly different in MDD and DDI from L2 academic writings with different language family backgrounds, which can be confirmed by the results of Mann–Whitney U test reported in Table 9, as the *p* values of the comparison of most pairs between English native academic writings and L2 academic writings are below significance level (0.05). One exception is the pair of Indo-European_Native vs. Pidgin-Creole in Arts & Humanities (*p* = 0.0503), showing no significant difference. This insignificance arises probably because pidgins and creoles are hybrid languages formed by the blending of different languages. For example, a pidgin language is one with vocabulary “of English, French, Spanish, or Portuguese origin” (Katzner, 2002: 32). As a result, it may share syntactical and lexical features with English, which in turn can influence the pidgin speakers’ English L2 academic writing. On the whole, a significant difference exists in MDD and DDI between English native academic writings and English L2 academic writings.

**Table 7:** MDD and DDI of samples from arts & humanities grouped by language family.

Language Family	Number of abstracts	Tokens (total)	Tokens (mean)	MDD	MDD (sd)	DDI	DDI (sd)
Indo-European_Native	454,861	81,825,987	179.9	2.9185	0.3493	0.6783	0.0363
Indo-European	255,527	48,960,196	191.6	2.9286	0.3489	0.6838	0.0358
Sino-Tibetan	38,485	7,262,799	188.7	2.9738	0.3522	0.6896	0.0365
Afro-Asiatic	24,341	4,673,168	192.0	2.9042	0.3813	0.6804	0.0353
Austronesian	17,329	3,611,675	208.4	2.8372	0.3388	0.6805	0.0334
Uralic	13,124	2,474,798	188.6	2.9242	0.3454	0.6884	0.0360
Altaic	9,506	1,892,020	199.0	2.9380	0.3466	0.6860	0.0353
Independent_Japanese	8,379	1,622,280	193.6	2.9616	0.3553	0.6885	0.0366
Niger-Congo	7,124	1,477,862	207.4	2.8946	0.3479	0.6751	0.0331
Independent_Korean	7,112	1,421,729	199.9	2.9538	0.3402	0.6887	0.0344
Tai	2,757	567,983	206.0	2.9292	0.3634	0.6847	0.0338
Mon-Khmer	1,286	249,436	194.0	2.9065	0.3495	0.6821	0.0347
Caucasian	387	72,489	187.3	2.9585	0.3326	0.6865	0.0367
Pidgin-Creole	132	26,791	203.0	2.9660	0.3143	0.6855	0.0322

**Table 8:** MDD and DDI of samples from social sciences grouped by language family.

Language Family	Number of abstracts	Tokens (total)	Tokens (mean)	MDD	MDD (sd)	DDI	DDI (sd)
Indo-European_Native	1,046,287	185,979,580	177.8	2.8950	0.3505	0.6782	0.0363
Sino-Tibetan	134,448	26,907,979	200.1	2.9836	0.3665	0.6948	0.0360
Indo-European	89,961	18,757,964	208.5	2.9155	0.3629	0.6813	0.0351
Independent_Korean	26,172	5,210,346	199.1	2.9435	0.3435	0.6913	0.0339
Independent_Japanese	23,012	4,619,206	200.7	2.9594	0.3551	0.6898	0.0358
Tai	9,198	1,985,591	215.9	2.9376	0.3866	0.6833	0.0339
Afro-Asiatic	6,266	1,325,606	211.6	2.9232	0.3597	0.6812	0.0350
Mon-Khmer	4,263	882,930	207.1	2.9214	0.3585	0.6833	0.0323
Niger-Congo	3,347	744,612	222.5	2.9233	0.3219	0.6717	0.0343
Austronesian	3,226	686,844	212.9	2.9224	0.3424	0.6810	0.0326
Uralic	2,559	527,516	206.1	2.9255	0.3339	0.6816	0.0369
Altaic	1,812	359,683	198.5	2.9161	0.3294	0.6836	0.0354
Caucasian	172	35,129	204.2	2.9823	0.3865	0.6754	0.0429
Pidgin-Creole	116	26,465	228.1	2.9528	0.3012	0.6751	0.0331

**Table 9:** Results of Mann–Whitney U test of MDD for samples grouped by language family.

Discipline	Language Family 1	Language Family 2	p	R
Arts & Humanities	Indo-European_Native	Austronesian	0.0000	−0.1552
	Indo-European_Native	Sino-Tibetan	0.0000	0.0976
	Indo-European_Native	Independent_Japanese	0.0000	0.0807
	Indo-European_Native	Indo-European	0.0000	0.0172
	Indo-European_Native	Independent_Korean	0.0000	0.0653
	Indo-European_Native	Niger-Congo	0.0000	−0.0514
	Indo-European_Native	Afro-Asiatic	0.0000	−0.0279
	Indo-European_Native	Altaic	0.0000	0.0333
	Indo-European_Native	Caucasian	0.0082	0.0776
	Indo-European_Native	Uralic	0.0210	0.0118
	Indo-European_Native	Pidgin-Creole	0.0503	0.0984
	Indo-European_Native	Mon-Khmer	0.0082	0.0776
	Indo-European_Native	Tai	0.0006	−0.0335
Social Sciences	Indo-European_Native	Afro-Asiatic	0.0000	0.0437
	Indo-European_Native	Indo-European	0.0000	0.0333
	Indo-European_Native	Niger-Congo	0.0000	0.0634
	Indo-European_Native	Sino-Tibetan	0.0000	0.1484
	Indo-European_Native	Uralic	0.0000	0.0699
	Indo-European_Native	Austronesian	0.0000	0.0492
	Indo-European_Native	Caucasian	0.0045	0.1251
	Indo-European_Native	Pidgin-Creole	0.0172	0.1277
	Indo-European_Native	Independent_Japanese	0.0000	0.1113
	Indo-European_Native	Altaic	0.0010	0.0445
	Indo-European_Native	Independent_Korean	0.0000	0.0861
	Indo-European_Native	Tai	0.0000	0.0506
	Indo-European_Native	Mon-Khmer	0.0000	0.0360

**Figure 6:**
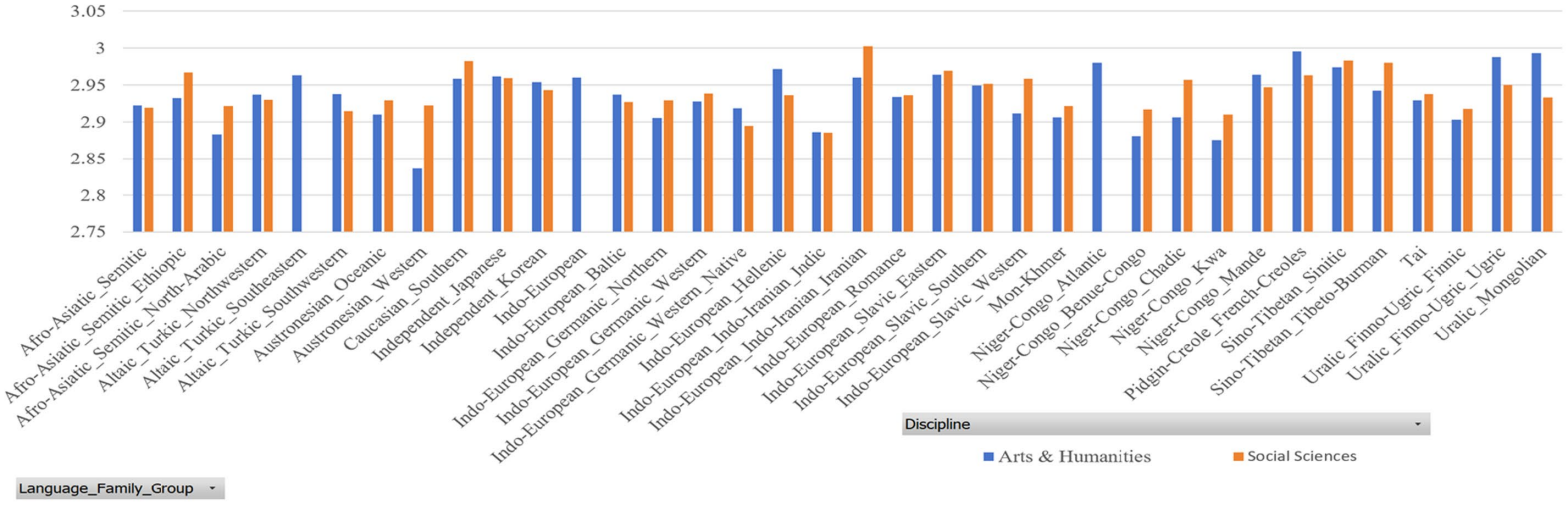
MDD of samples grouped by language family group.

**Figure 7:**
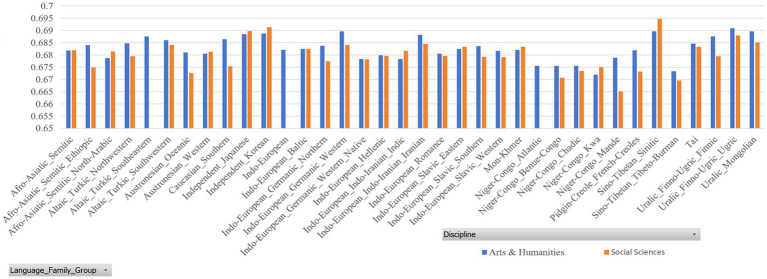
DDI of samples grouped by language family group.

Similar results in Figures 6, 7 are found between English native academic writings and English L2 academic writings grouped by language family group. (Detailed reports of the MDD and DDI are not presented here and are available upon request as they take much space). A significant difference arises between English native and L2 academic writings, which is confirmed by the significant p values, which is below 0.05. (Detailed reports of the Mann–Whitney U test are available upon request to save space here).

We also grouped the English L2 academic writings according to whether English is regarded as an official language of the countries where these L2 writings are from. As Table 10 shows, English L2 academic writings with a background of non-English official languages have a longer MDD and higher ratio of DDI, with a significant difference (*p* < 0.05) from the English native academic writings as Table 11 shows. For those L2 writings with English as the official language of their countries, no significant difference (*p* = 0.5798) from English native academic writings is found for samples from Social Sciences, though a significant difference is found for those from the Arts and Humanities.

**Table 10:** MDD and DDI of samples grouped by type of official language.

Discipline	Official_ Language	Number of abstracts	Tokens (total)	Tokens (mean)	MDD	MDD (sd)	DDI	DDI (sd)
Arts & Humanities	Native	454,861	81,825,987	179.9	2.9185	0.3493	0.6783	0.0363
	Non_English	349,878	67,123,651	191.8	2.9327	0.3523	0.6849	0.0359
	English	35,667	7,201,983	201.9	2.8824	0.3430	0.6787	0.0341
Social Sciences	Native	1,046,287	185,979,580	177.8	2.8950	0.3505	0.6782	0.0363
	Non_English	245,988	49,950,349	203.1	2.9654	0.3597	0.6905	0.0359
	English	58,658	12,141,307	207.0	2.8982	0.3720	0.6812	0.0351

**Table 11:** Results of Mann–Whitney U test of MDD for samples grouped by official language.

Discipline	Language 1	Language 2	p	R
Arts & Humanities	Native	Non_English	0.0000	0.0243
	Native	English	0.0000	−0.0691
	Native	Native	1.0000	0.0000
Social Sciences	Native	Non_English	0.0000	0.1196
	Native	Native	1.0000	0.0000
	Native	English	0.5798	−0.0014

## 6 Discussion

Native language transfer has been found in the learning and use of a second language among learners of different language backgrounds (for example, Madrid, 1981; Gerlach, 2017; Rios Castaño, 2021; Chai and Bao, 2023). However, few studies have examined whether the dependency relations of English L2 academic writings are influenced by native language transfer effects, even though scholars like Siu and Ho (2015) have explored the transfer of syntactic features in bilingual students. Our study of the English native and L2 academic writings within the disciplines of Arts and Humanities and Social Sciences finds a significant difference in their MDD and DDI. Significant differences in both MDD and DDI are typically observed between the native group and L2 subgroups, regardless of whether L2 academic writings are analyzed as a whole, from different language backgrounds, or from various language families. The significant difference is especially explicit when the MDDs of native English and the native languages of English L2 academic writings are significantly different. Take Chinese, Hungarian, and German, which belong to Sino-Tibetan, Uralic, and Indo-European families respectively, for example, the three languages have a much longer MDD (3.662, 3.446, 3.353) than native English (2.543). The L2 academic writings with the background of the three languages also have a much longer MDD than that of the English native academic writings (2.9896, 2.9464, and 2.9500 vs. 2.8950). For another example, when looked at from the perspective of word order typology, a significant difference is also found between native English (SVO) and the native languages (SOV) of English L2 academic writings, like Korean, which mainly falls into the type of SOV word order and is regarded by some linguists as a language in the Altaic family (Brown and Ogilvie, 2009: 250). The MDD of English L2 academic writings with Korean as a native language is much longer than English native academic writings (2.9435 vs. 2.8950). English L2 academic writings with a background of native language being Spanish, which “tends to prefer an OVS order” (Brown and Ogilvie, 2009: 884), also have a much longer MDD than that of English native academic writings (2.9400 vs. 2.8950). The MDD of Spanish is also longer than native English (2.665 vs. 2.543). Besides, in terms of DDI, English native academic writings are also significantly different from English L2 academic writings as a whole or from different language backgrounds or different language families. The majority of English L2 academic writings have a higher ratio of DDI than English native academic writings.

Drawing on the discussed findings, the first two research questions can now be addressed. It can be confidently stated that English L2 academic writings exhibit significant differences in MDD and DDI compared to English native academic writings. The greater the MDD in their native languages, the longer the MDD tends to be in the English L2 academic writings.

To address the third question, which is central to this study, we conducted regression and correlation analyses as previously discussed. These analyses investigate the potential relationship between the dependent variable (predicted) and independent (predictor) variable. In the regression analysis, the findings indicate that MDD and DDI, when used as predictor variables, successfully predict the outcomes, clearly distinguishing between native and L2 academic writings. Likewise, the results from the correlation analysis reveal that the MDD and DDI are significantly correlated to the dependent variable, distinguishing between native and L2 academic writings. Why English L2 academic writings are different in MDD and DDI from English native academic writings? Despite being published in similar or identical journals, these academic writings are authored by scholars from diverse language backgrounds: both native and non-native English speakers. For non-native English speakers, their academic writings exhibit characteristics typical of L2 texts, influenced by the phenomenon of native language transfer, as identified in previous research. The disparities in MDD and DDI between English L2 and native academic writings are likely attributed to the effect of native language transfer. This influence is particularly pronounced in L2 academic writings from background languages with a significantly longer MDD compared to native English, resulting in a substantially extended MDD in these texts.

However, native language transfer might not be the sole factor influencing the MDD of English L2 academic writings. For instance, we observe that English L2 academic writings with a Japanese language background exhibit a significantly longer MDD compared to English native academic writings (2.9595 vs. 2.8950), despite the fact that the MDD of Japanese itself is considerably shorter than that of native English (1.805 vs. 2.543). A plausible explanation for this phenomenon could be that, alongside native language transfer, other factors such as interlanguage interference (Antoniou et al., 2011) also play a significant role. This observation suggests that native language is one of the factors influencing the dependency relations of English L2 academic writings.

## 7 Conclusions and implications

Through a large dataset of English abstracts from the disciplines of Arts and Humanities and Social Sciences, the present study investigates the dependency distance and direction to examine whether native language influences the MDD of English L2 academic writings. It is found that English L2 and native academic writings differ significantly from each other in MDD and DDI. The regression and correlation analyses reveal that native language tends to be a factor influencing the MDD of English L2 academic writings. The greater the MDD of the native languages compared to that of native English, the longer the MDD in English L2 academic writings relative to English native academic writings. However, for languages with MDDs that are not significantly greater than that of native English, while the MDDs of English L2 and native academic writings differ significantly, the MDDs of English academic writings are not necessarily longer than those of English native academic writings. This observation suggests that additional factors, such as interlanguage interference, also influence the MDD of English L2 academic writings.

The findings could provide implications for both L2 academic writing and instruction. To increase the readability of their English academic writings, English L2 writers could try to make their writings similar to English native academic writings in MDD. For example, L2 writers from languages with significantly longer MDD than English must overcome L1 transfer effects to reduce the MDD in their English L2 academic writings. For writing instruction, the findings highlight the necessity of teaching students about the varying patterns of dependency relations between their native language and English, to make them aware of the different norms of MDD in their native language and in English. Specifically, the findings, which reveal that English L2 writings of different L1 backgrounds exhibit systematically different dependency profiles reflective of L1 transfer, underscore the value of conducting contrastive analysis between L1 and L2, as well as the importance of L1-focused instruction in academic writing pedagogy. Tailored syllabuses, targeted exercises, and native language scaffolds could be developed to help particular L1 groups reduce negative transfer effects. For example, in teaching L2 writers hailing from Chinese, Hungarian, German, and Spanish backgrounds, it is beneficial to focus on raising their consciousness to lower the MDD in English writings. Given that these languages have a significantly greater MDD than English, they have a more substantial influence on the transfer of dependency relations. This goal can be accomplished by contrasting the syntactic norms of their native languages with English, with a special emphasis on the varying patterns of dependency relations.

Though our study examined the MDD of English L2 writings through a large dataset, the datasets are mainly academic writings from the disciplines of Arts and Humanities and Social Sciences. Whether datasets from other disciplines or genres will yield similar or the same results is yet to be confirmed. Besides, in answering the question of whether native language influences the MDD of English L2 academic writings, we mainly rely on regression and correlation analyses. Though these analyses can reveal the causal relationship between the predictor variable MDD and DDI and the predicted variable article type (native vs. L2), it is not completely safe to conclude that native language influences the MDD of the English L2 academic writings of all language backgrounds, because there are no statistics of the MDDs of different native languages. If there are enough statistics of these MDDs in the regression and correlation analyses, it will be convincing to draw such a conclusion as more direct influence and correlation can be revealed through the analysis. Future studies could probably confirm our findings by including the MDDs of different native languages in the analysis. Besides, as our data is very large, it is unavoidable that there might be some abstracts that are not completely clean even though we have made several rounds of data cleaning. Nevertheless, the majority of our data are well-cleaned, the few unclean data do not affect the findings of our study.

## Data availability statement

The original contributions presented in the study are included in the article/supplementary material, further inquiries can be directed to the corresponding author.

## Author contributions

YB: Conceptualization, Supervision, Writing – review & editing. HT: Data curation, Resources, Software, Visualization, Writing – original draft.
